# A case of gallstone pancreatitis with a history of choledochojejunostomy treated by the endoscopic ultrasound rendezvous technique and stone removal from the residual bile duct

**DOI:** 10.1055/a-2513-2605

**Published:** 2025-01-28

**Authors:** Fumiya Kataoka, Mitsuru Okuno, Atsushi Tagami, Hiroshi Araki, Eiichi Tomita, Hisataka Moriwaki, Masahito Shimizu

**Affiliations:** 1 73505 Department of Gastroenterology, Matsunami General Hospital, Gifu, Japan; 2 First Department of Internal Medicine, Gifu University Hospital, Gifu, Japan

Gallstone pancreatitis necessitates emergency endoscopic removal of the responsible stone 1 . We report a case highlighting three significant clinical characteristics: 1) recurrent stone in the residual bile duct of the pancreatic head 13 years after choledochojejunostomy; 2) a challenging transpapillary approach to the buried papilla within a diverticulum; and 3) use of the endoscopic ultrasound rendezvous (EUS-RV) technique to access the bile duct. Successful stone removal alleviated the pancreatitis ( Video 1 ).

Removal of a common bile duct stone from the residual bile duct in the pancreatic head using the endoscopic ultrasound rendezvous technique for gallstone pancreatitis in a patient with a history of choledochojejunostomy.Video 1

An 80-year-old man presented with abdominal pain. He had undergone choledochojejunostomy and cholecystectomy 13 years previously to remove common bile duct (CBD) stones and gallbladder stones. Computed tomography revealed a CBD stone in the residual bile duct of the pancreatic head and peripancreatic fluid collection ( Fig. 1 **a** ). Endoscopic retrograde cholangiography was attempted for management of the gallstone pancreatitis; however, the orifice of the main papilla could not be located owing to the intradiverticular papilla ( Fig. 2 ). The rendezvous technique was used to access the CBD. Given the history of choledochojejunostomy and post-cholecystectomy, EUS-RV via the intrapancreatic CBD was considered the sole curative method for CBD stone removal ( Fig. 1 **b** ).

**Fig. 1:**
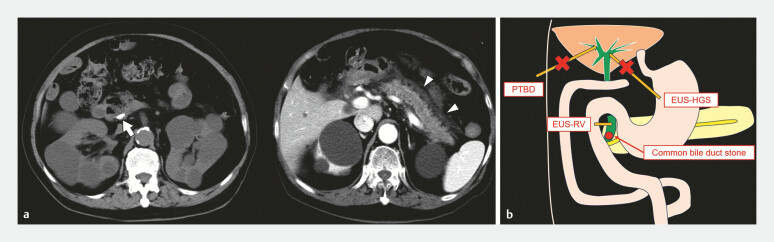
Computed tomography (CT) findings during initial admission and diagram of the patient’s anatomy. **a** An 8-mm common bile duct (CBD) stone was detected (arrow), with accompanying peripancreatic fluid accumulation noted (arrowhead) on CT. **b** After choledochojejunostomy, the percutaneous transhepatic biliary drainage and endoscopic ultrasound (EUS)-guided hepaticogastrostomy routes could not access the residual bile duct. EUS-guided hepaticogastrostomy via the intrapancreatic CBD was therefore considered the sole curative method for removing the CBD stone. EUS-HGS, endoscopic ultrasound-guided hepaticogastrostomy; PTBD, percutaneous transhepatic biliary drainage; EUS-RV, endoscopic ultrasound rendezvous.

**Fig. 2:**
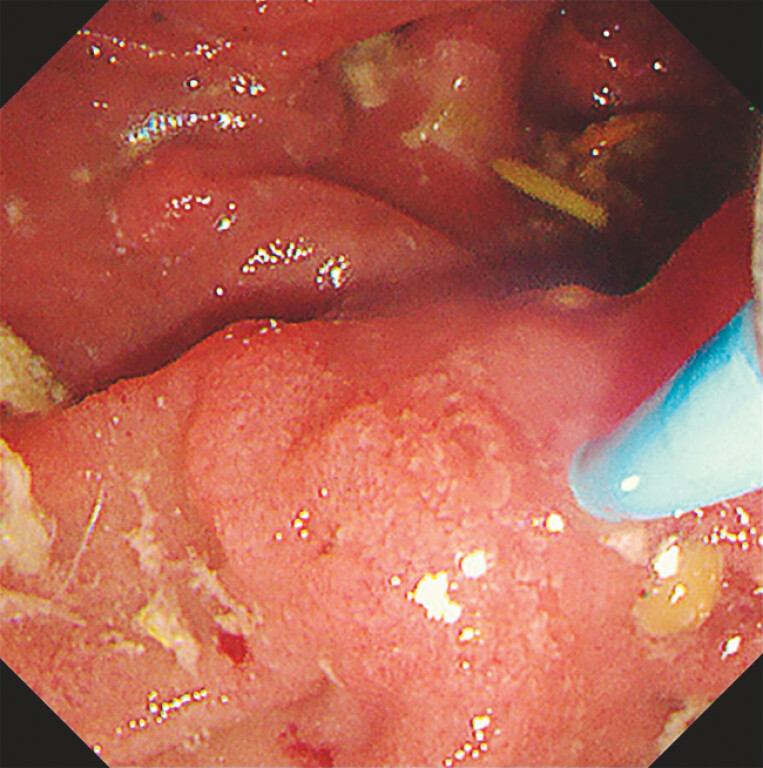
Endoscopic findings from the initial endoscopic retrograde cholangiography procedure. The orifice of the main papilla could not be located owing to the presence of an intradiverticular papilla.

After EUS (UCT-260; Olympus Medical Systems, Tokyo, Japan) revealed an 8-mm CBD stone ( Fig. 3 **a** ), the intrapancreatic CBD was punctured via the pancreas using a 19-G needle (EZ Shot 3 Plus; Olympus Medical Systems) ( Fig. 3 **a** ). A 0.025-inch guidewire was then inserted into the CBD and advanced into the duodenum via the main papilla. We switched to a duodenoscope (TJF-Q290V; Olympus Medical Systems) and successfully cannulated the CBD after grasping the guidewire. The CBD stone was subsequently removed after endoscopic papillary large-balloon dilation ( Fig. 3 **b–d** , Video 1 ).

**Fig. 3:**
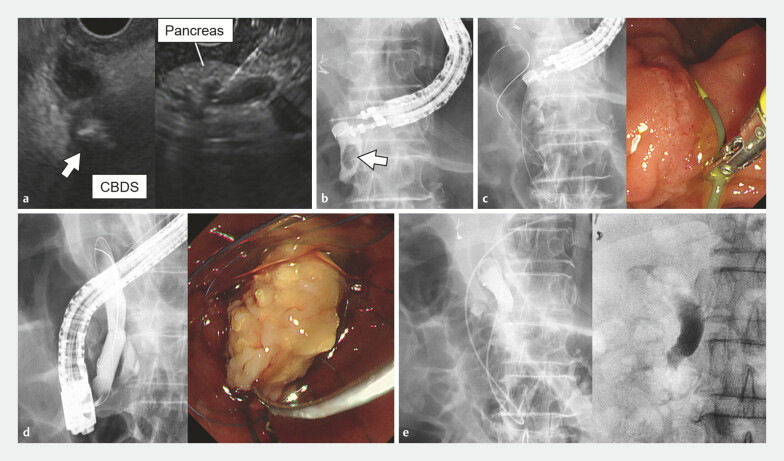
Common bile duct (CBD) stone removal using the endoscopic ultrasound (EUS) rendezvous technique. **a** EUS showed an 8-mm CBD stone in the intrapancreatic CBD (arrow). The CBD was punctured through the pancreas. **b** Cholangiography after CBD puncture confirmed the presence of the CBD stone (arrow). **c** The guidewire was advanced into the duodenum via the papilla. **d** Successful cannulation of the CBD was achieved using the guidewire. The CBD stone was ultimately removed after endoscopic papillary large-balloon dilation. **e** After the procedure, an endoscopic nasobiliary drainage tube was placed in the CBD to prevent pancreatic juice leakage. The drainage tube was removed 3 days later, after confirming the absence of pancreatic leakage. CBDS, common bile duct stone.

As EUS-RV required puncturing through the pancreatic parenchyma, we placed an endoscopic nasobiliary drainage tube in the CBD to prevent leakage of pancreatic juice ( Fig. 3 **e** ). No adverse events were observed, and the pancreatitis improved after the procedure.

Endoscopy_UCTN_Code_TTT_1AS_2AH

## Notes

Endoscopy E-Videos https://eref.thieme.de/e-videos
*E-Videos* is an open access online section of the journal *Endoscopy* , reporting on interesting cases and new techniques in gastroenterological endoscopy. All papers include a high-quality video and are published with a Creative Commons CC-BY license. Endoscopy E-Videos qualify for HINARI discounts and waivers and eligibility is automatically checked during the submission process. We grant 100% waivers to articles whose corresponding authors are based in Group A countries and 50% waivers to those who are based in Group B countries as classified by Research4Life (see: https://www.research4life.org/access/eligibility/ ). This section has its own submission website at https://mc.manuscriptcentral.com/e-videos .
